# Preferential interaction of MHC class I with TAPBPR in the absence of glycosylation

**DOI:** 10.1016/j.molimm.2018.06.269

**Published:** 2019-09

**Authors:** Andreas Neerincx, Louise H. Boyle

**Affiliations:** Department of Pathology, University of Cambridge, Tennis Court Road, Cambridge, CB2 1QP, UK

**Keywords:** Antigen processing and presentation, Tapasin, TAPBPR/TAPBPL, MHC, N-linked glycosylation

## Abstract

We recently discovered that TAPBPR promotes reglucosylation of the N-linked glycan on MHC class I molecules, a modification that restores their recognition by calreticulin and reincorporation into the peptide-loading complex. We wondered whether TAPBPR displayed some degree of glycan specificity, as is known to be the case for tapasin via its interaction with calreticulin & ERp57, or whether its interaction with MHC class I was glycan independent. Here, we explored this by comparing the ability of TAPBPR to bind to MHC class I containing either an intact or disrupted NxS/T glycosylation consensus sequence. In contrast to tapasin, TAPBPR bound strongly to MHC class I molecules that lacked N-linked glycosylation, suggesting that the TAPBPR:MHC class I interaction is glycan independent. Furthermore, we found that glycosylated HLA-A2 preferentially interacts with tapasin rather than TAPBPR, possibly explaining, in part, why MHC class I molecules bind efficiently to tapasin in the face of an alternative chaperone. The distinction in glycan specificity between the two peptide editors suggests that TAPBPR may bind to MHC class I molecules that are associated with a broader diversity of oligosaccharides attached compared with tapasin. This may explain, to some extent, the ability of TAPBPR to interact with MHC class I molecules outside of the ER.

## Highlights

• TAPBPR interacts with MHC class I in a glycan independent manner.
• In contrast to TAPBPR, the association of tapasin with MHC class I is glycan dependent.
• Distinct conformations of MHC class I are recognised by the two peptide editors.
• Non-glycosylated MHC class I preferentially bind to TAPBPR rather than tapasin.

## Graphical abstract

**Figure:**
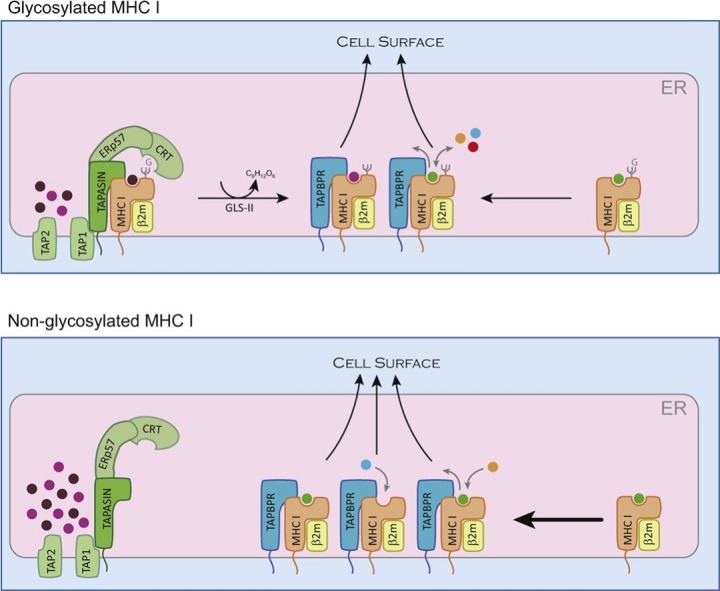

## 1 Introduction

It is well established that MHC class I molecules perform a critical role in infection control and tumour recognition by presenting antigenic peptides to CD8^+^T lymphocytes. Precisely how MHC class I molecules determine which peptides to present for immune recognition remains somewhat enigmatic in comparison. In the mid-1990 s, tapasin was identified as a molecular bridge between MHC class I and the TAP transporters (Sadasivan et al., 1996; Li et al., 1997; Ortmann et al., 1997). Subsequently, extensive research has demonstrated an important role for tapasin in peptide selection onto MHC class I, not only by bringing peptide-receptive MHC class I close to source of newly degraded peptide, but also by functioning as an MHC class I peptide editor, a process that helps improve the affinity of peptides bound to MHC class I molecules (Williams et al., 2002; Howarth et al., 2004; Chen and Bouvier, 2007; Wearsch and Cresswell, 2007). More recently, we discovered TAPBPR is a second MHC class I dedicated chaperone in the antigen presentation pathway, which exhibits peptide exchange functionality (Boyle et al., 2013; Hermann et al., 2015). A role for TAPBPR in promoting peptide editing has similarly been found by Margulies and colleagues (Morozov et al., 2016). Although TAPBPR only shares 22% identity with tapasin (Teng et al., 2002), we found that some of the MHC class I binding sites are shared between the two chaperones, resulting in similar orientation on MHC class I (Hermann et al., 2013). However, there are a number of key differences between tapasin and TAPBPR, as recently reviewed (Neerincx and Boyle, 2017). One crucial distinction is differences in their association partners. For example, while tapasin is a key component of the peptide loading complex (PLC) forming interactions with TAP and ERp57 (Sadasivan et al., 1996; Li et al., 1997; Ortmann et al., 1997; Dick et al., 2002; Peaper et al., 2005), TAPBPR is not (Boyle et al., 2013). This difference likely has a significant influence with respect to how these two peptide exchange catalysts shape the repertoire of peptides presented by MHC class I molecules.

Recently, we investigated whether any additional co-factors associate with TAPBPR and found that it binds to UDP-glucose:glycoprotein glucosyltransferase (UGT1) (Neerincx et al., 2017). This enzyme serves an important quality control role in the ER and cis-Golgi through the regeneration of the Glc_1_Man_9_GlcNAc_2_ moiety on incompletely folded or unassembled glycoproteins, thereby restoring recognition by calnexin and calreticulin (Tannous et al., 2015). A critical role of UGT1 in MHC class I quality control was demonstrated through the observation that it reglucosylates the N-linked glycan on MHC class I molecules associated with suboptimal peptides causing their PLC reengagement and that optimal assembly, peptide selection and surface expression of MHC class I was impaired in UGT1-deficient cells (Wearsch et al., 2011; Zhang et al., 2011). Our findings suggest that in addition to functioning as a peptide editor, TAPBPR also acts as a bridge between UGT1 and MHC class I, thereby promoting the reglucosylation of the glycan on peptide receptive class I/β2 m heterodimers, which restores their recognition by calreticulin and consequently their reengagement with the PLC (Neerincx et al., 2017).

Glycosylation serves several important functional roles in the life of a protein including determining structure and stability, monitoring quality control and regulating egress to the cell surface. Human MHC class I molecules have a single conserved N-linked glycan on an asparagine residue found at position 86 (Parham et al., 1977). The functional relevance of glycosylation of human MHC class I has been explored using a number of approaches including mutation of the NxS/T motif to eliminate N-linked glycosylation (Barbosa et al., 1987; Santos-Aguado et al., 1987; Zhang et al., 1995; Harris et al., 2001; Martayan et al., 2008; Rizvi et al., 2011). Non-glycosylated human MHC class I molecules fail to interact with calreticulin, exhibit weak interaction with tapasin and the PLC, and are consequently expressed at lower levels at the cell surface. Although tapasin is known to bind to MHC class I in a glycosylation dependent manner via its interactions with ERp57 and calreticulin (Radcliffe et al., 2002; Wearsch and Cresswell, 2008; Del Cid et al., 2010; Wearsch et al., 2011), the glycan dependency of the interaction between TAPBPR and MHC class I has yet to be investigated. This is of particular interest in light of our recent findings that TAPBPR functions in cooperation with UGT1 in MHC class I quality control. Our aim here was to determine whether the interaction of TAPBPR with MHC class I was dependent on the N-linked glycan on MHC class I.

## 2 Materials and methods

### 2.1 Plasmids

The production of PK1-A2, which contains a generic N-terminal signal sequence, a GFP cassette followed by a myc tag, a GAGA linker and the sequence corresponding to exon 2–8 of HLA-A2 and untagged HLA-A2 in the lentiviral vector pHRSINcPPT-SGW has previously been described (Boyle et al., 2006; Hermann et al., 2013). Mutation of N86Q and S88R was achieved via site directed mutagenesis using the following primers: A2-N86Q-for 5′-GGGACCCTGCGCGGCTACTACCAACAGAGCGAGGCCGGTTCTCACAC-3′, A2-N86Q-rev 5′-GTGTGAGAACCGGCCTCGCTCTGTTGGTAGTAGCCGCGCAGGGTCCC-3′, A2-S88R-for 5′-GCGCGGCTACTACAACCAGAGAGAGGCCGGTTCTCACACCGTC-3′, A2-S88R-rev 5′-GACGGTGTGAGAACCGGCCTCTCTCTGGTTGTAGTAGCCGCGC-3′. Untagged HLA-A*68:02, HLA-A*68:02^N86Q^, HLA-A*68:02^S88R^, HLA-B*27:05^N86Q^ and HLA-B27:05^S88R^ were kindly provided by Tudor Ilca (University of Cambridge, UK). Knockout of the classical MHC class I molecules was achieved using sgRNAs specific for HLA-A (sgRNA-A1:ACAGCGACGCCGCGAGCCAG), HLA-B (sgRNA-B2:GGTTCTATCTCCTGCTGGTC) and HLA-C (sgRNA-C2: CGGACTGGTCATACCCGCGG), initially described in Shalem et al. (2014) cloned into pSpCas9 (BB)-2 A-puro (Kind gifts from Liye Chen and Paul Bowness, University of Oxford, UK). Knockout of TAPBPR was achieved using the sgRNA sequence GCGAAGGACGGTGCGCACCG cloned into pSpCas9 (BB)-2 A-puro as previously described (Hermann et al., 2015).

### 2.2 Cell lines, transfection and transduction

HeLa-M, a variant HeLa cell line that is more responsive to IFN (Tiwari et al., 1987), and 293 T (both kind gifts from Paul Lehner, University of Cambridge, UK) were maintained in Dulbecco’s modified eagle’s medium (DMEM)(SIGMA) supplemented with 10% fetal calf serum, 100 U/ml penicillin and 100 μg/ml streptomycin at 37 °C and 5% C0_2_. Cell lines were confirmed to be mycoplasma negative using the MycoAlert kit from Lonza. To induce TAPBPR expression and upregulate expression of other components of the antigen processing and presentation pathway cells were treated with 200 U/ml human IFN-γ (Peprotech) for 48–72 h. Lentiviral transduction of cells was performed as previously described (Neerincx et al., 2017). The generation of HeLa-M knockout cell lines using CRISPR was performed using the method previously described (Hermann et al., 2015). To select HeLa-M cells lines deficient in classical MHC class I expression single cell cloning was performed after transfection with the three HLA-A, -B and -C sgRNAs containing plasmids. HLA-A,-B, -C knockout cell lines were screened using flow cytometry.

### 2.3 Antibodies

The mouse mAb PeTe4 raised against amino acids 22–406 of human TAPBPR (confirmed not to cross-react with tapasin), was used to isolate TAPBPR via immunoprecipitation (Boyle et al., 2013; Hermann et al., 2013). Ab57411 (Abcam, UK), a mouse mAb raised against amino acids 23–122 of TAPBPR was used to detect TAPBPR by western blot. The tapasin-specific mAb Pasta 1 (Dick et al., 2002) and the rabbit polyclonal antibody R.gp48N (Sadasivan et al., 1996) (Both kind gifts from Peter Cresswell, Yale University School of Medicine, New Haven, CT) were used to detect tapasin via immunoprecipitation and western blot analysis, respectively. ab290 (Abcam, UK), a rabbit polyclonal specific for GFP and the mouse anti-GFP 11 814 460 001 (Roche), were used to detect GFP via immunoprecipitation and western blot analysis, respectively. The mAb W6/32, which has broad specificity for HLA-A, -B, -C was used to detect HLA class I associated with β2 m and peptide (Barnstable et al., 1978). The mAb BB7.2 was used to detect peptide loaded and β2 m bound HLA-A2 (Parham and Brodsky, 1981). The mouse mAbs HCA2, which recognises HLA molecules containing xLxTLRGx at residues 77–84 (Stam et al., 1990; Sernee et al., 1998) and HC10, which recognises HLA molecules containing PxxWDR at residues 57–62 (Stam et al., 1986; Perosa et al., 2003) and the rat monoclonal antibody 3B10.7 which exhibits broad specificity for HLA class I (Lutz and Cresswell, 1987) were used to detect MHC class I via western blot analysis. Heavy chain/β2 m heterodimers of HLA-A68 and HLA-A2 were detected using BIH0037 from One Lambda (Thermo Fisher Scientific, Canoga Park, CA). HLA-B molecules were detected on HeLa-M cells using the mAb 4E (Yang et al., 1984). HLA-C and –E were detected using DT9 (Braud et al., 1998). Calnexin was detected using the rabbit polyclonal ADI-SPA-860 (Enzo Life Sciences, UK). The rabbit mAb ab124879 (Abcam) was used to detect UGT1. Isotype control antibodies (Dako, UK), horseradish peroxidase (HRP)-conjugated species-specific secondaries (Dako and Rockland Immunochemicals Inc., Limerick, PA), and species-specific Alexa-Fluor secondary antibodies (Invitrogen Molecular Probes Thermo Fisher Scientific) were also used.

### 2.4 Flow cytometry

HeLa-M cells were detached from flasks using trypsin, followed by washing in 1% bovine serum albumin (BSA)/PBS at 4 °C. Cells were stained at 4 °C for 25 min in 1% BSA/PBS with the indicated antibodies or with an isotype control antibody. After washing the cells to remove excess unbound antibody, primary antibodies bound to the cells were subsequently detected by incubation at 4 °C for 25 min with goat anti-mouse Alexa-Fluor 647 or Strep-A647 (Invitrogen Molecular Probes, Thermo Fisher Scientific). Samples were read on a BD FACScan analyser with Cytek modifications and analysed using FlowJo (FlowJo, LLC, Ashland, OR).

### 2.5 Immunoprecipitation and Western blot analysis

Harvested cells were washed in PBS, then lysed in 1% digitonin (Calbiochem, Merck millipore) in Tris buffer saline (TBS) (20 mM Tris−HCl, 150 mM NaCl, 2.5 mM CaCl_2_) supplemented with 10 mM *N*-ethylmaleimide (Sigma), 1 mM phenylmethylsulfonyl fluoride and protease inhibitor cocktail (Roche) for 30 min. at 4 °C. Nuclei and cell debris were removed by centrifugation and supernatants were subsequently precleared on IgG-Sepharose (GE Healthcare) and protein A–Sepharose (Generon, UK) beads. Immunoprecipitations were performed using the indicated Ab and protein A–Sepharose for 2–3 h at 4 °C with rotation, followed by thorough washing in 0.1% digitonin TBS. Samples were subsequently heated at 95 °C for 10 min in reducing sample buffer (125 mM Tris−HCl pH 6.8, 4% SDS, 20% glycerol, 0.04% bromophenol blue supplemented with 100 mM β-mercaptoethanol). Proteins were separated using SDS-PAGE followed by transfer onto Immobilon transfer membranes (Merck Millipore). Membranes were blocked using 5% (w/v) dried milk in PBS with 0.1% (v/v) Tween 20 (PBS/Tween) for 30 min. Membranes were subsequently incubated with the indicated primary antibody for 1–16 h, washed extensive in PBS/Tween then incubated with species-specific HRP-conjugated secondary antibodies for 1 h. Following washing in PBS/Tween, reactive bands were detected by enhanced chemiluminescence using Western Lightning (Perkin Elmer, UK) and Super RX film (Fujifilm, UK) or Syngene G:BOX Chemi XRQ.

### 2.6 Pulse chase analysis

Cells were starved for 30 min in methionine-free, cysteine-free RPMI medium, labelled using EasyTag Express [^35^S]-protein labelling mix (PerkinElmer) for 20 min, then chased in medium supplemented with 3 mM unlabelled l-methoinine (Sigma) and 60 μM unlabelled cysteine (Sigma) all at 37 °C. Samples taken at 0, 20, 45 and 90 min post-pulse, were subsequently washed in cold PBS, and lysed in 1% digitonin/TBS at 4 °C. Immunoprecipitation of tapasin and TAPBPR were performed as described above. Denatured samples were separated using SDS-PAGE. Gels were subsequently fixed in 12% acetic acid, 40% methanol and dried. Images were obtained using a phosphor screen (Perkin-Elmer) and on film. PhosphorImager analysis was performed using Typhoon Trio variable mode imager (GE Healthcare) together with ImageQuantTL software.

## 3 Results

### 3.1 The N-linked glycan on MHC class I is required for efficient surface expression of GFP-A2

**Fig. 1:**
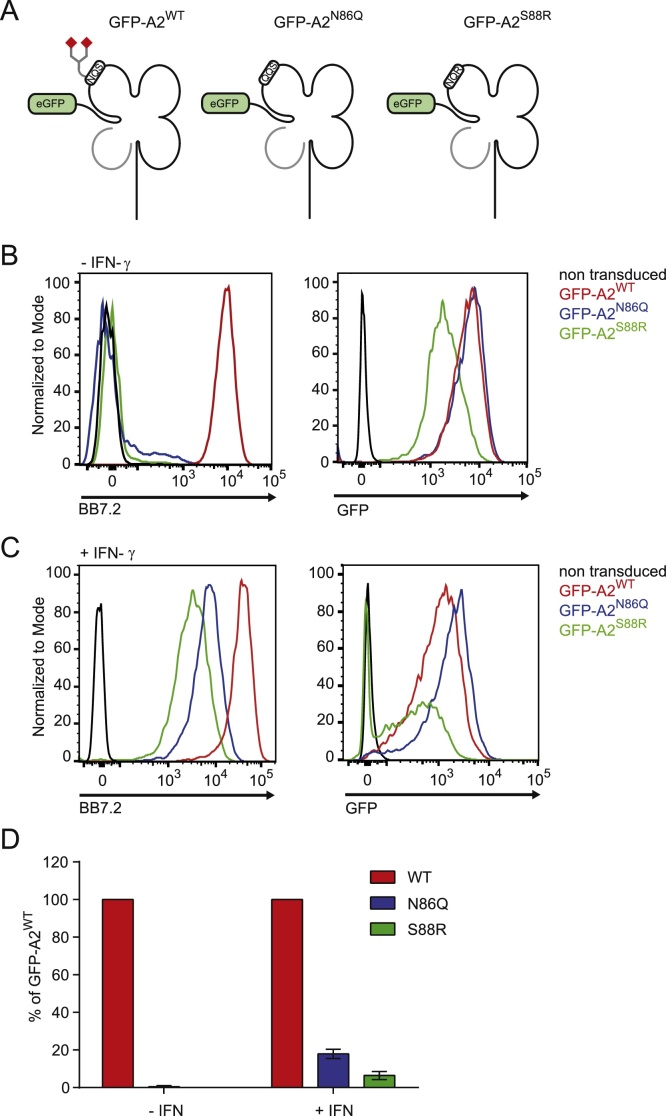
The N-linked glycan on MHC class I is required for efficient surface expression of GFP-A2. (A) Cartoon representation of the N-terminally GFP tagged HLA-A2 molecules used in this study. GFP-A2^WT^ contains the consensus sequence that permits glycosylation of HLA-A2, whereas GFP-A2^N86Q^ and GFP-A2^S88R^ are non-glycosylated due to disruption of the motif. (B & C) Surface expression of GFP-A2 detected with the conformational specific mAb BB7.2 and total GFP expression to demonstrate transduction efficiency in HeLa-M cells expressing GFP-A2^WT^ (red line), GFP-A2^N86Q^ (blue line) and GFP-A2^S88R^ (green line) in (B) the absence and (C) the presence of IFN-γ treatment for 48 h. Staining of non-transduced HeLa-M cell with BB7.2 is included as a control (black line).The results are representative of three independent experiments. (D) Bar graphs showing mean fluorescence intensity of surface HLA-A2 expression on the panel of GFP-A2 expressing cell lines as a percentage of GFP-A2^WT^. Error bars represent SEM from three independent experiments as performed in B & C. (For interpretation of the references to color in this figure legend, the reader is referred to the web version of this article.)

To determine if the interaction between TAPBPR and MHC class I was dependent on the glycan found on residue 86, we initially used HLA-A2 molecules tagged at the N-terminus with GFP (Boyle et al., 2006) (Fig. 1A). We created HLA-A2 glycosylation mutants by disrupting the NxS/T motif using in two independent approaches; one was by mutating the asparagine residue found a position 86 to glutamine (A2^N86Q^), the other was by mutating the serine residue at position 88 to arginine (A2^S88R^) (Fig. 1A). When this panel of GFP-tagged HLA-A2 molecules was expressed in HeLa-M cells, we observed high expression of GFP-A2^WT^ on the cell surface as detected with the HLA-A2 conformational specific mAb BB7.2 (Fig. 1B). In contrast, surface expression of both GFP-A2^N86Q^ and GFP-A2^S88R^ was very low at the cell surface (Fig. 1B). Analysis of total GFP expression in the cells revealed a similar transduction efficiency for GFP-A2^WT^ and GFP-A2^N86Q^, but lower (although still significant) expression of GFP-A2^S88R^ in comparison to GFP-A2^WT^ (Fig. 1B). Therefore, the lack of detection of GFP-A2^N86Q^ and GFP-A2^S88R^ at the cell surface was not due to a lack of protein expression of these two molecules. Upon IFN-γ treatment, which induces TAPBPR expression in HeLa cells (Boyle et al., 2013) and also increases expression of other components involved the antigen processing and presentation, the surface expression of both GFP-A2 glycan mutants was significantly increased (Fig. 1C). However, surface expression of GFP-A2^N86Q^ and GFP-A2^S88R^ were significantly lower compared with GFP-A2^WT^, being at only 20% or less of that of GFP-A2^WT^ (Fig. 1D). The defect in surface expression we observed with the HLA-A2 glycosylation mutants is in keeping with other published findings for HLA-A2 and other human HLA molecules lacking the glycan at position 86 (Barbosa et al., 1987; Santos-Aguado et al., 1987; Harris et al., 2001; Martayan et al., 2008; Rizvi et al., 2011).

### 3.2 The interaction of TAPBPR with GFP-A2 occurs in a glycosylation independent manner

**Fig. 2:**
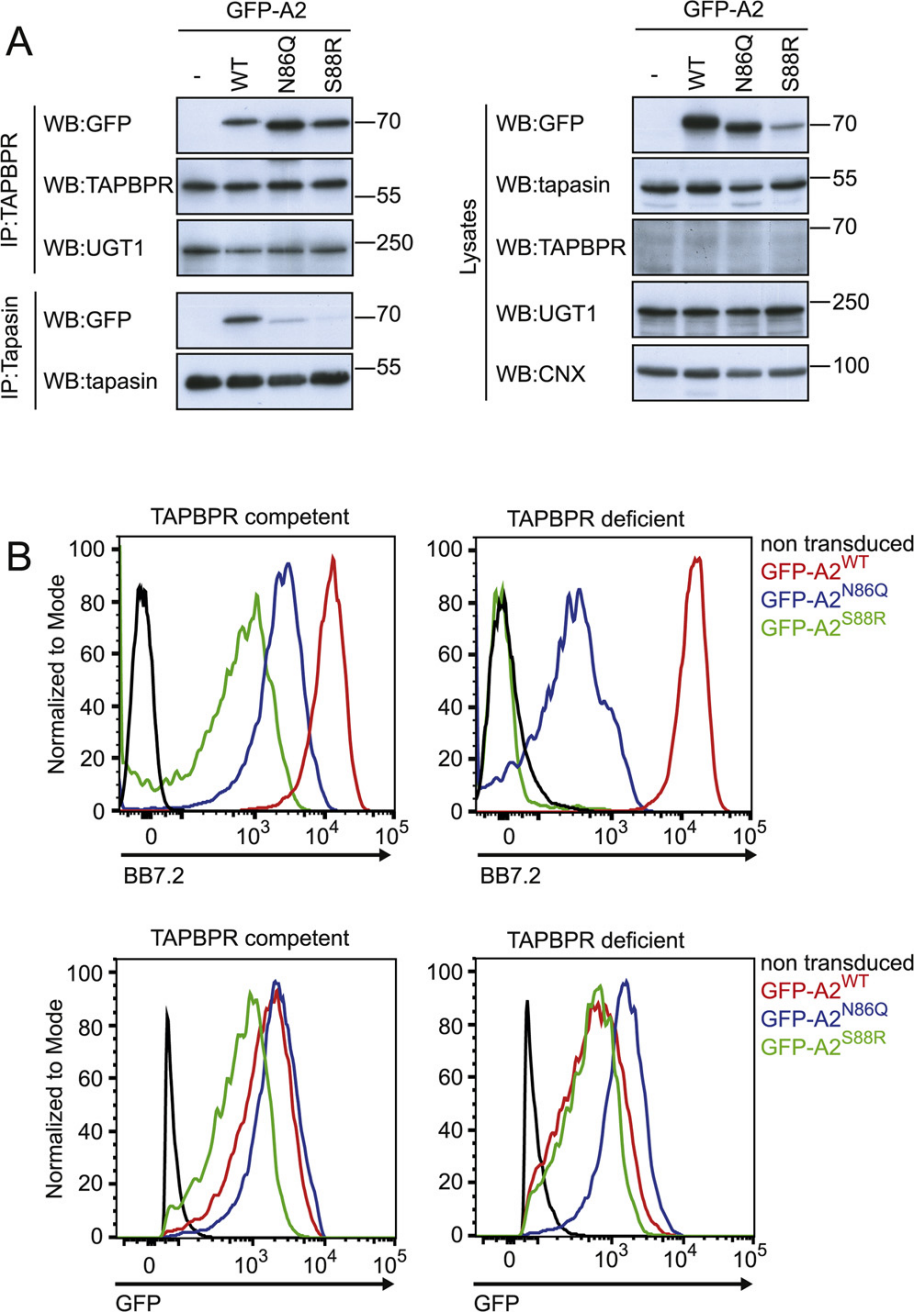
The interaction of TAPBPR with GFP-A2 occurs in a glycosylation independent manner. (A) TAPBPR and tapasin were immunoprecipitated from IFN-γ treated HeLa-M cells expressing the panel of GFP-A2 molecules. Non-transduced cells were included as a negative control. Western blot analysis was performed for GFP, TAPBPR, tapasin, UGT1 or calnexin (CNX) on immunoprecipitates and lysates as indicated. Additional repeats are shown in supplementary Fig. 1. (B) Surface expression of GFP-A2 detected using the conformational specific mAb BB7.2 on IFN-γ treated HeLa-M cells (TAPBPR competent) and IFN-γ treated HeLa-M cells with TAPBPR knocked out (TAPBPR deficient) expressing GFP-A2^WT^ (red line), GFP-A2^N86Q^ (blue line) and GFP-A2^S88R^ (green line). Staining of non-transduced HeLa-M cell with BB7.2 is included as a control (black line). Only GFP positive cells were included in the analysis using BB7.2. The GFP levels on cells gated for GFP is shown in the lower panel. The results are representative of three independent experiments. (For interpretation of the references to color in this figure legend, the reader is referred to the web version of this article.)

To determine if the interaction between TAPBPR and MHC class I was dependent on the glycan found on residue 86, TAPBPR was immunoprecipitated from the panel of GFP-A2 expressing cells after IFN-γ treatment. TAPBPR interacted with both the glycosylated (WT) and the non-glycosylated (N86Q and S88R) forms of GFP-A2 (Fig. 2A). This suggests that the interaction between TAPBPR and MHC class I is not dependent on the glycan found at position 86 on MHC class I. In fact our results suggest that the association between TAPBPR and GFP-A2 may be stronger in the absence of glycosylation, given that the total cellular protein levels of GFP-A2^S88R^ was lower than GFP-A2^WT^, but TAPBPR interacted equally well with both these proteins (Fig. 2A). In contrast, the interaction between tapasin and MHC class I was decreased in the absence of glycosylation of the GFP-A2 (Fig. 2A).

### 3.3 Surface expression of non-glycosylated GFP-A2 is dependent on TAPBPR

Given the strong association observed for TAPBPR with GFP-A2^N86Q^ and GFP-A2^S88R,^ but the weak association seen for both these molecules with tapasin, we next determined whether surface expression of these non-glycosylated molecules was dependent on TAPBPR. We compared the surface expression of the panel of GFP-A2 molecules in wild-type HeLa-M cells with TAPBPR-deficient HeLa-M cells treated with IFN-γ (see (Hermann et al., 2015) for characterisation of this cell line). Surface expression GFP-A2^WT^ was similar in TAPBPR-competent and TAPBPR-deficient HeLa-M cells (Fig. 2B). This is in keeping with our previous observations with HLA-A2 (Hermann et al., 2015), and suggests the steady state surface expression of GFP-A2^WT^ is not significantly affected by TAPBPR expression. In contrast, surface expression of both GFP-A2^N86Q^ and GFP-A2^S88R^ was significantly decreased in TAPBPR-deficient HeLa-M cells (Fig. 2B). These data suggest that glycosylation deficient GFP-A2 molecules are dependent on TAPBPR for their surface expression.

### 3.4 Glycosylation of N86 is required for efficient surface expression of untagged HLA-A2

**Fig. 3:**
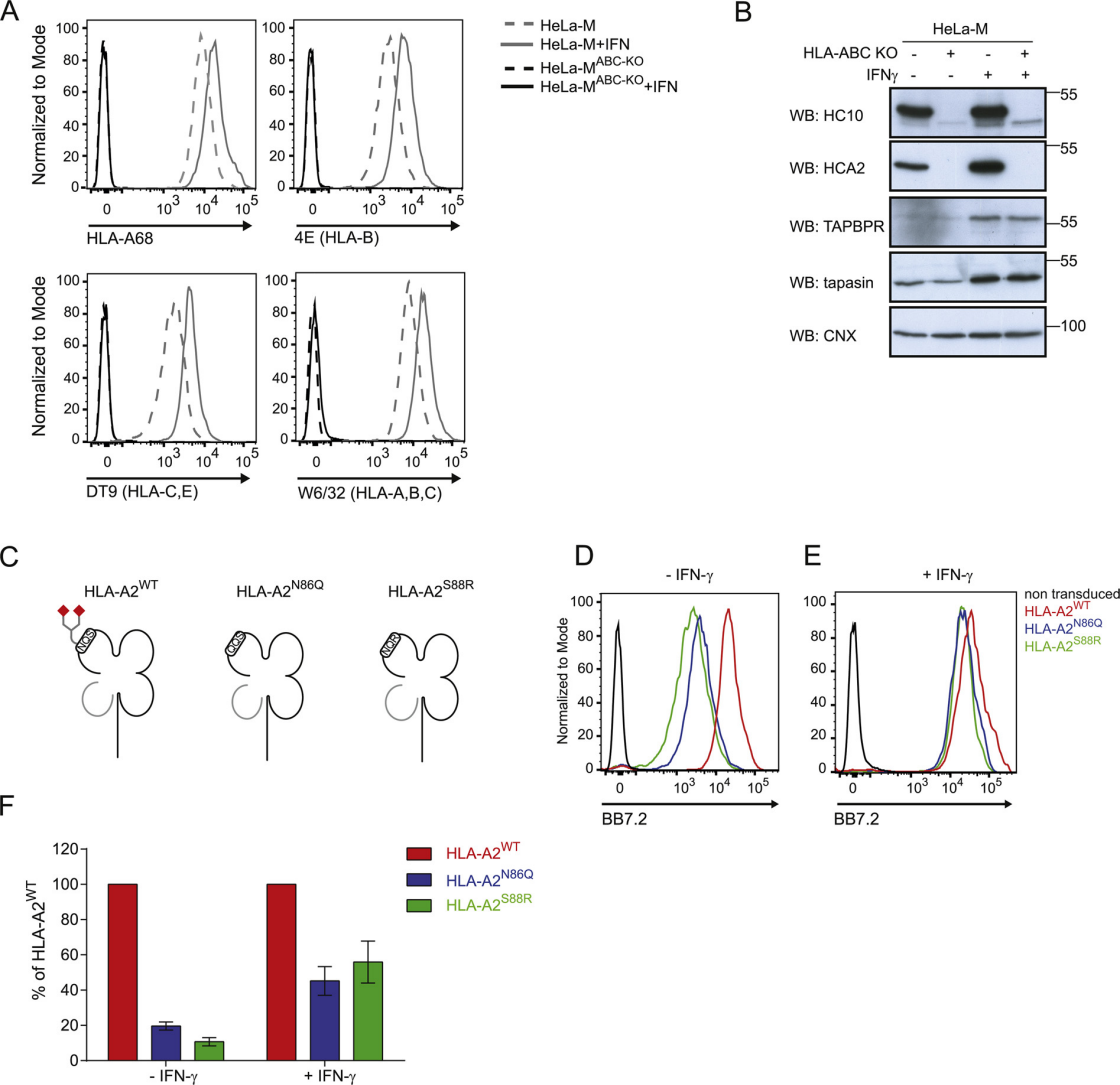
Glycosylation of N86 is required for efficient surface expression of untagged HLA-A2. (A) Surface expression of HLA-A68, HLA-B (using 4E), HLA-C and –E (using DT9) and pan-MHC class I (detected with W6/32) on HeLa-M (grey line) and HeLa-M^ABC−KO^ cells (black line) treated without (dashed line) and with IFN-γ for 48 h (solid line). (B) Western blot analysis of MHC class I (detected with HC10 and HCA2), TAPBPR, and tapasin on HeLa-M and HeLa-M^ABCKO^ cells treated without and with IFN-γ for 48 h. Blotting with calnexin is included as a loading control. (C) Cartoon representation of untagged HLA-A2 molecules transduced into the HeLa-M^ABC-KO^ cells. HLA-A2^WT^ contains the consensus sequence that permits glycosylation of HLA-A2, whereas HLA-A2^N86Q^ and HLA-A2^S88R^ are non-glycosylated due to disruption of the motif. (D&E) Surface expression of untagged HLA-A2 detected with the conformational specific mAb BB7.2 on HeLa-M^ABC−KO^ cells expressing HLA-A2^WT^ (red line), HLA-A2^N86Q^ (blue line) and HLA-A2^S88R^ (green line) in (D) the absence and (E) the presence of IFN-γ treatment for 48 h. Staining of non-transduced HeLa-M^ABC-KO^ cells is included as a control (black line). (F) Bar graphs showing mean fluorescence intensity of surface HLA-A2 expression on the panel of HLA-A2 expressing HeLa-M^ABC-KO^ cells as a percentage of HLA-A2^WT^. Error bars represent SEM from three independent experiments as performed in D & E. (For interpretation of the references to color in this figure legend, the reader is referred to the web version of this article.)

Although the results with GFP-tagged HLA-A2 suggested that TAPBPR was capable of interacting strongly with non-glycosylated HLA-A2 and that surface expression of these molecules was dependent on TAPBPR, we felt it was important to confirm these findings using untagged HLA-A2 molecules, to mitigate the potential influence of the GFP tag. In order to proceed with the investigations in a similar cellular background, without interference or competition from endogenous HLA molecules, we created a HeLa cell line lacking expression of HLA-A, -B and -C using CRISPR-Cas9. Flow cytometric and western blot analysis confirmed that our selected HeLa-M cell line lacked HLA-A, -B and C expression (HeLa-M^ABC−KO^), both at the cell surface (Fig. 3A) and at a total protein level (Fig. 3B**)**, even following IFN-γ treatment. We then transfected the HeLa-M^ABC-KO^ cells with untagged HLA-A2^WT^, HLA-A2^N86Q^ or HLA-A2^S88R^ (Fig. 3C). The three HLA-A2 variants were well expressed on the cell surface in the absence of IFN-γ with as detected BB7.2 (Fig. 3D). However, HLA-A2^WT^ surface expression was significantly higher than either of the two glycosylation mutant molecules. Although the attenuation of cell surface expression of non-glycosylated HLA-A2 compared to wild-type molecules observed with the untagged molecules (Fig. 3D) generally correlated with cell surface expression patterns observed using the GFP-tagged HLA-A2 variants (Fig. 1B), a more severe decrease in cell surface expression was observed upon mutation of the NxS/T motif when HLA-A2 was tagged with GFP (Fig. 1B). Following IFN-γ treatment, surface expression of all untagged HLA-A2 molecules increased (Fig. 3E). However, surface expression of HLA-A2^N86Q^ and HLA-A2^S88R^ remained lower compared to HLA-A2^WT^ (Fig. 3E & F). Therefore, glycosylation of N86 appears to be required for optimal surface expression of untagged HLA-A2.

### 3.5 In contrast to tapasin, TAPBPR binds to non-glycosylated HLA-A2

**Fig. 4:**
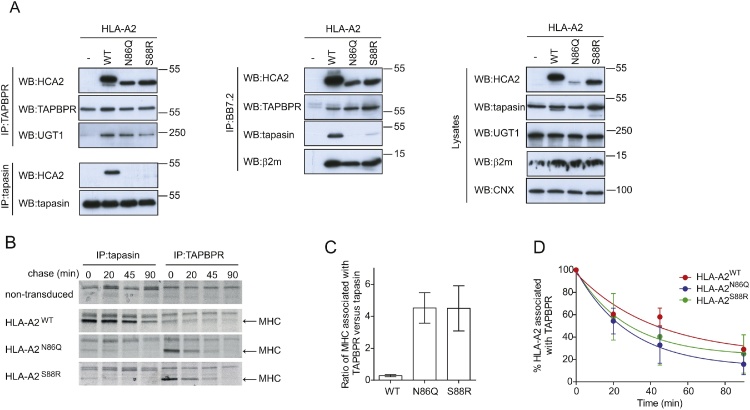
In contrast to tapasin, TAPBPR binds to non-glycosylated HLA-A2. (A) TAPBPR, tapasin and HLA-A2 (using BB7.2) were immunoprecipitated from IFN-γ treated HeLa-M^ABC−KO^ cells expressing the panel of HLA-A2 molecules. Non-transduced cells were included as a negative control. Western blot analysis was performed for the HLA heavy chains using HCA2, TAPBPR, tapasin, UGT1, β2 m or calnexin (CNX) on immunoprecipitates and lysates as indicated. An additional repeat is shown in supplementary Fig. 2. (B) IFN-γ treated HeLa-M^ABC−KO^ cells expressing the panel of HLA-A2 molecules were labelled for 20 min with [^35^S] cysteine/methionine, chased for the indicated time followed by immunoprecipitation for tapasin or TAPBPR. (C) Bar graph shows the ratio of newly synthesised HLA-A2 molecules associated with TAPBPR as compared to tapasin at the 0 time point as performed in B. (D) Graph shows the percentage of HLA-A2 associated with TAPBPR over time as a percentage of the signal at the 0 time point as performed in B. The results in C and D are from duplicate experiments. Error bar = -/+SEM.

To characterise the interactions of tapasin and TAPBPR with the glycosylated and non-glycosylated forms of HLA-A2, the two chaperones were immunoprecipitated from IFN-γ treated HeLa-M^ABC−KO^ cells expressing the panel of untagged HLA-A2 molecules. Western blot analysis revealed that TAPBPR interacted efficiently with HLA-A2^WT^ and the two glycosylation mutants HLA-A2^N86Q^ and HLA-A2^S88R^ (Fig. 4A). These results suggest that the interaction between TAPBPR and MHC class I is not dependent on the glycan found at position 86 on MHC class I. In contrast, tapasin interacted strongly with HLA-A2^WT^ but did not interact efficiently with HLA-A2^N86Q^ or HLA-A2^S88R^ (Fig. 4A). These results suggest that the interaction of tapasin with MHC class I is dependent on the N-linked glycosylation of the heavy chain at position 86. These findings are generally consistent with our results using the GFP-tagged HLA-A2 molecules (Fig. 2). Immunoprecipitation of BB7.2 reactive HLA-A2, further confirmed the interaction of glycosylated HLA-A2 with both tapasin and TAPBPR, the lack of association of non-glycosylated HLA-A2 with tapasin and the strong binding of non-glycosylated HLA-A2 with TAPBPR (Fig. 4A). Furthermore, in the BB7.2 immununoprecipiates there appeared to be more TAPBPR associated with the HLA-A2 glycan mutants compared to wild-type molecules, despite the fact that less BB7.2 reactive material was isolated for the glycan mutants (Fig. 4A).

### 3.6 In the absence of glycosylation MHC class I molecules preferentially interact with TAPBPR

Given our previous findings regarding the role of the TAPBPR:UGT1 complex in reglucosylating MHC class I molecules, which consequently recycles them back to the PLC (Neerincx et al., 2017), we were intrigued to determine the fate of non-glycosylated MHC class I and wondered whether they remained retained on TAPBPR in the absence of glycosylation. Using pulse chase analysis, we compared the rates of association of newly synthesised glycosylated and non-glycosylated HLA-A2 with tapasin and TAPBPR. This revealed that newly synthesised glycosylated HLA-A2 preferentially interacted with tapasin over TAPBPR (Fig. 4B&C). In contrast, upon removal of the N-linked glycan the newly synthesised HLA-A2 preferentially interacted with TAPBPR rather than tapasin (Fig. 4B&C). However, the non-glycosylated HLA-A2 dissociated from TAPBPR over time (Fig. 4B&D), suggesting HLA-A2^N86Q^ and HLA-A2^S88R^ were not retained on TAPBPR.

### 3.7 TAPBPR also binds strongly to non-glycosylated HLA-A*68:02 and HLA-B*27:05

**Fig. 5:**
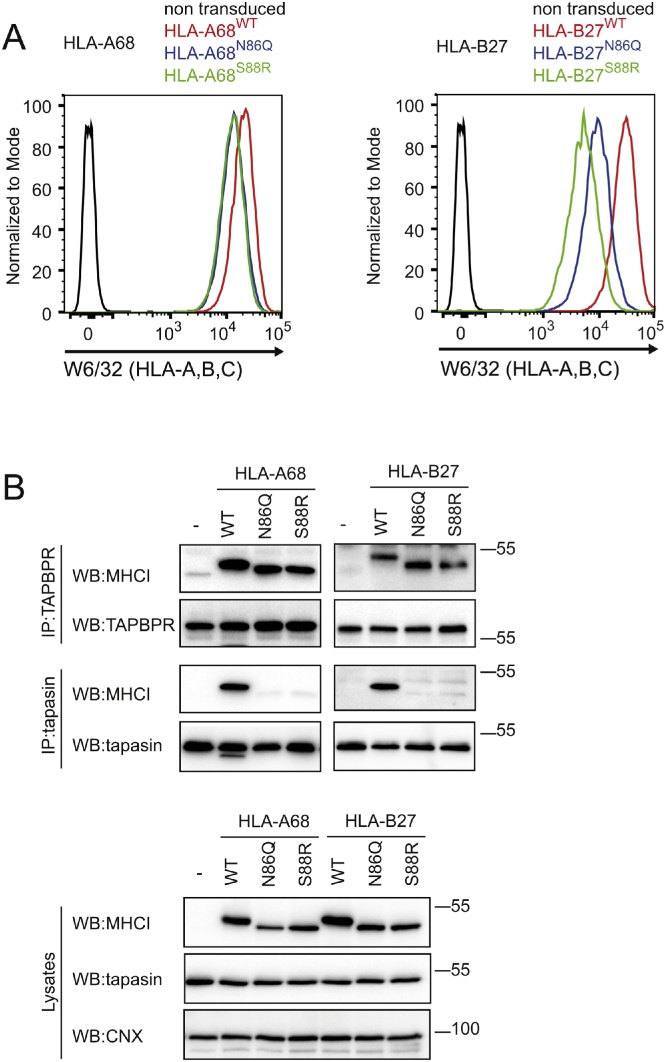
TAPBPR binds to non-glycosylated HLA-A*68:02 and HLA-B*27:05. (A) Surface expression of untagged HLA-A*68:02 and HLA-B*27:05, detected with W6/32, on HeLa-M^ABC−KO^ cells expressing WT (red line), N86Q (blue line) and S88R (green line) HLA variants in the absence of IFN-γ treatment. Staining of non-transduced HeLa-M^ABC-KO^ cells with W6/32 is included as a control (black line). (B) TAPBPR and tapasin were immunoprecipitated from IFN-γ treated HeLa-M^ABC−KO^ cells expressing the panel of HLA-A*68:02 and –B*27:05 molecules. Non-transduced cells were included as a negative control. Western blot analysis was performed for the HLA heavy chains, TAPBPR, tapasin, or calnexin (CNX) on immunoprecipitates and lysates as indicated. The results are representative of three independent experiments. Addition repeats are shown in supplementary Fig. 3. (For interpretation of the references to color in this figure legend, the reader is referred to the web version of this article.)

Next, we determined the importance of glycosylation in mediating TAPBPR binding to two other MHC class I molecules, HLA-A*68:02 and HLA-B*2705. When transduced into HeLa-M^ABC−KO^ cells, glycan-deficient variants of these two additional alleles were expressed at lower levels than their wild-type counterparts (Fig. 5A). While tapasin only bound to wild-type HLA-A*68:02 and HLA-B*2705, TAPBPR bound strongly to both wild-type and glycan-deficient variants of these two additional MHC class I molecules (Fig. 5B). Although both non-glycosylated HLA-A*68:02 and –B*27:05 were expressed at lower steady state levels than wild-type molecules, the glycan-deficient variants associated with TAPBPR to a similar extent as their WT counterparts (Fig. 5B). This suggests that non-glycosylated untagged HLA molecules may bind more strongly to TAPBPR than the WT HLA molecules. Thus, TAPBPR can bind to HLA-A*68:02, -B*2705 as well as –A*02:01 in both the presence and absence of the attached glycan, suggesting that the TAPBPR:MHC class I interaction is glycan-independent.

### 3.8 Surface expression of non-glycosylated HLA-A2 is lower in the absence of TAPBPR

**Fig. 6:**
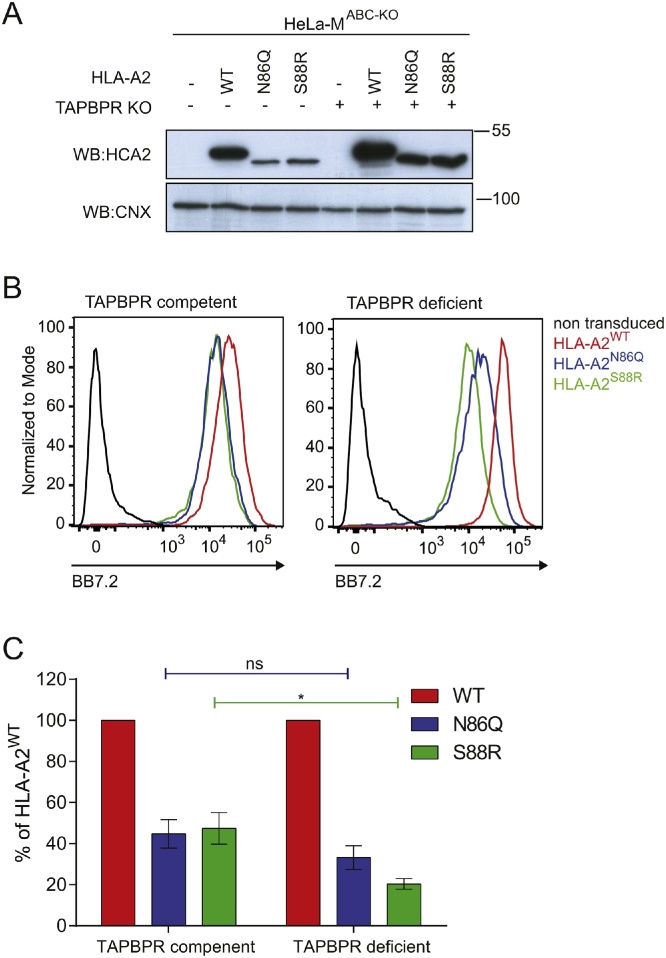
Surface expression of non-glycosylated HLA-A2 is slightly lower in the absence of TAPBPR. (A) Western blot analysis of HLA-A2 expression (detected with HCA2) on IFN-γ treated HeLa-M^ABC−KO^ without (-) and with TAPBPR knocked out (+). Blotting with calnexin (CNX) is included as a loading control. (B) Surface expression of HLA-A2 detected using the conformational specific mAb BB7.2 on IFN-γ treated HeLa-M^ABC−KO^ cells (TAPBPR competent) and IFN-γ treated HeLa-M^ABC−KO^ cells with TAPBPR knocked out (TAPBPR deficient) expressing HLA-A2^WT^ (red line), HLA-A2^N86Q^ (blue line) and HLA-A2^S88R^ (green line). Staining of non-transduced HeLa-M^ABC−KO^ cells with BB7.2 is included as a control (black line). The results are representative of three independent experiments. (C) Bar graphs showing mean fluorescence intensity of surface HLA-A2 expression on IFN-γ treated HeLa-M^ABC−KO^ cells (TAPBPR competent) and IFN-γ treated HeLa-M^ABC−KO^ cells with TAPBPR knocked out (TAPBPR deficient) as a percentage of HLA-A2^WT^. Error bars represent SEM from three independent experiments as performed in B. (For interpretation of the references to color in this figure legend, the reader is referred to the web version of this article.)

Given the strong association of untagged HLA-A2^N86Q^ and HLA-A2^S88R^ with TAPBPR, we determined whether surface expression of these non-glycosylated molecules was dependent on TAPBPR. As performed previously for the GFP-A2 molecules in Fig. 2B, we compared the surface expression of the panel of HLA-A2 molecules in wild-type HeLa-M^ABC−KO^ cells with TAPBPR knockout HeLa-M^ABC−KO^ cells treated with IFN-γ. Western blot analysis suggested the transduction efficiency was higher in the TAPBPR-deficient HeLa-M^ABC−KO^ cells compared to the TAPBPR-competent cells as the total amount of HLA-A2 heavy chain detected for all three untagged HLA molecules was higher (Fig. 6A). However, as observed in the TAPBPR-competent cells, we observed lower expression levels of the glycosylation mutants compared to HLA-A2^WT^ in TAPBPR-deficient cells (Fig. 6A). Surface expression of untagged HLA-A2^WT^ was similar in TAPBPR-competent and TAPBPR-deficient HeLa-M cells (Fig. 6B). In contrast, surface expression of both HLA-A2^N86Q^ and HLA-A2^S88R^ was slightly lower in the TAPBPR-deficient HeLa-M^ABC−KO^ cells compared to TAPBPR-competent HeLa-M^ABC−KO^ (Fig. 6B&C), despite the increase in the steady state expression of the HLA-A2 molecules in the TAPBPR-deficient cells. Although, the decrease observed for HLA-A2^N86Q^ did not reach significance, the decrease observed in surface expression for HLA-A2^S88R^ upon TAPBPR depletion was statistically significant (Fig. 6C). Together, these results suggest that in the absence of glycosylation HLA-A2 molecules become more dependent on TAPBPR for efficient surface expression.

## 4 Discussion

Modification of the glycan attached to the MHC class I heavy chain is important for its efficient folding, peptide loading and quality control. Although the glycan dependency of tapasin for human MHC class I has previously been investigated (Harris et al., 2001; Radcliffe et al., 2002; Martayan et al., 2008; Del Cid et al., 2010; Rizvi et al., 2011; Wearsch et al., 2011), this has not yet been characterised for TAPBPR. We recently found that TAPBPR can influence the oligosaccharide attached to MHC class I by working with UGT1 (Neerincx et al., 2017). Consequently, TAPBPR helps send peptide-receptive MHC class I molecules back into the calnexin/calreticulin pathway, which causes their re-association with tapasin (Neerincx et al., 2017). Therefore, we were intrigued as to whether TAPBPR displayed some degree of glycan specificity, for example, binding to MHC class I molecules with sugar moieties other than the Glc_1_Man_9_GlcNAc_2,_ which tapasin preferentially interacts with via its interaction with ERp57 and calreticulin (Radcliffe et al., 2002; Wearsch and Cresswell, 2008; Del Cid et al., 2010; Wearsch et al., 2011); or whether its interaction with MHC class I was glycan independent. Here, we explored this by determining whether TAPBPR is capable of interacting with HLA-A2 that lacks N-linked glycosylation, produced by disrupting the glycosylation consensus sequence NxS found at residues 86–88 of the MHC class I heavy chain.

We found that TAPBPR binds strongly to MHC class I molecules lacking an N-linked glycan. Therefore, and in contrast to tapasin, the interaction of TAPBPR with MHC class I appears to be glycan independent. Although tapasin and TAPBPR are orientated on MHC class I in a similar manner (Hermann et al., 2013), we have identified at least one obvious distinction in the conformation of MHC class I that the two chaperones are capable of binding.

Although our findings were generally consistent whether we used GFP-tagged HLA-A2 or untagged HLA-A2, one disparity observed was that the non-glycosylated GFP-tagged HLA-A2 molecules (Fig. 2B) appeared to be more dependent on TAPBPR for surface expression than non-glycosylated untagged HLA-A2 (Fig. 6C). Clearly, the GFP tag did not inhibit tapasin or TAPBPR binding to HLA-A2 and surface expression of the GFP-A2^WT^ protein was unaffected by this N-terminal modification. However, our results may suggest in the absence of glycosylation the GFP tag may inhibit the efficient folding and peptide loading of MHC class I, perhaps by inhibiting another chaperone from binding. It may also be worth noting that HLA-A2 molecules are known to be able to efficiently self-assemble with peptide in a tapasin independent manner (Greenwood et al., 1994), potentially explaining the high surface expression of the untagged, non-glycosylated HLA-A2 molecules, which are unable to bind to tapasin. Self-loading of HLA-A2 may also explain the lack of a severe phenotype observed on surface expression of the untagged, non-glycosylated HLA-A2 in TAPBPR deficient cells. With this in mind, the GFP tag may inhibit the ability of HLA-A2 to self-load, rendering these molecules more dependent on tapasin and TAPBPR for efficient surface expression.

Collectively, our observations provide several lines of evidence to suggest that the association of TAPBPR with MHC class I actually increases in the absence of the glycan. Firstly, in immunoprecipition:western blot experiments performed at steady state, we observed a strong interaction between non-glycosylated MHC class I molecules and TAPBPR even in conditions in which the glycan-deficient MHC class I are expressed at lower levels compared to glycosylated counterparts (Figs. 4A & 5B). Secondly, by pulse chase analysis, we observe that the preference of newly synthesised HLA-A2 for tapasin and TAPBPR swapped following glycan removal (Fig. 4B). A number of possibilities could explain these observations. For example, the stronger association between TAPBPR and MHC class I could be due to a lack of competition with tapasin. This would be consistent with our previous observation that the association between TAPBPR and newly synthesised MHC class I increases 2-fold in tapasin knockout cells (Hermann et al., 2013). In addition, our findings may be due to a substantial pool of the non-glycosylated MHC class I remaining in conformations favoured by TAPBPR, such as peptide receptive states or loaded with suboptimal peptides (Morozov et al., 2016). Alternatively, TAPBPR binding to MHC class I may be sterically hindered in the presence of Glc_1_Man_9_GlcNAc_2._

In light of the discovery of TAPBPR as a second peptide editor in the MHC class I antigen presentation pathway a number of questions have arisen. One question relates to the determinants that govern whether an MHC class I molecule interacts with tapasin or TAPBPR (i.e. how is the interaction of MHC class I with the two peptide editors controlled within the ER?). It is also unclear why two distinct peptide editors are required to select the peptide repertoire presented on MHC class I. The results presented here provide some insight into these fundamental questions.

Current data suggests that TAPBPR may exhibit a higher affinity for MHC class I than tapasin. Firstly, the luminal domains of TAPBPR alone can catalyse peptide exchange on MHC class I (Hermann et al., 2015; Morozov et al., 2016) whereas tapasin requires other cofactors or conjugation to MHC class I through a leucine zipper to perform peptide editing *in vitro* (Chen and Bouvier, 2007; Wearsch and Cresswell, 2007). Secondly, affinity studies suggest that TAPBPR has a higher affinity for at least some MHC class I allomorphs than tapasin (Morozov et al., 2016). Therefore, it initially seems surprising that tapasin can compete with TAPBPR at all in the ER. However, it is noteworthy that, to date, the *in vitro* studies performed for TAPBPR involved the use of MHC class I heavy chains produced in bacteria (Hermann et al., 2015; Morozov et al., 2016). Therefore, the MHC class I molecules utilised *in vitro* will naturally lack any N-linked glycosylation and the fact that TAPBPR functions on these non-glycosylated molecules adds further support our findings here in cells. It is possible, however, that the affinity observed between TAPBPR and MHC class I *in vitro* may be substantially lower *in vivo* when the heavy chain is glycosylated. Therefore, our findings here that glycosylated MHC class I preferentially bind to tapasin over TAPBPR may help explain how MHC class I molecules bind efficiently to tapasin in the face of alternative choice (although the alternative molecule can of course feed MHC class I back to tapasin) (Neerincx et al., 2017). Our results also shed light on the need for two chaperones/peptide editors in the antigen presentation pathway: one, tapasin, clearly has specificity for molecules with a Glc_1_Man_9_GlcNAc_2_ moiety attached via its interaction with calreticulin; the other, TAPBPR, interacts in a glycan independent manner, potentially permitting broader glycan specificity. Therefore, TAPBPR appears to permit monitoring and peptide editing of MHC class I molecules in alternative sites within the antigen presentation pathway.

## Funding

This work was funded by a Wellcome Trust Senior Fellowship (104647/Z/14/Z) held by LHB.

## Figures and Tables

**Figure:**
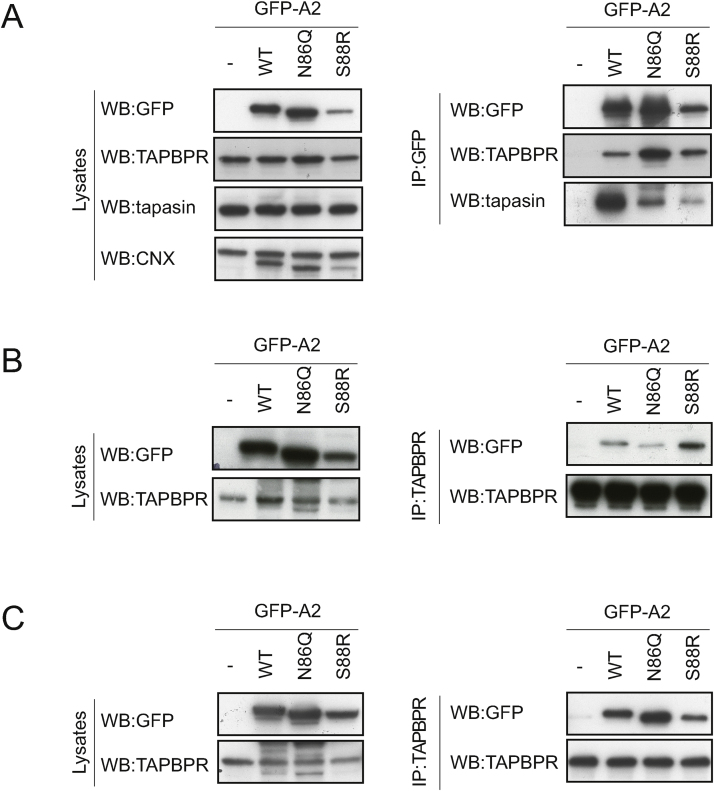


**Figure:**
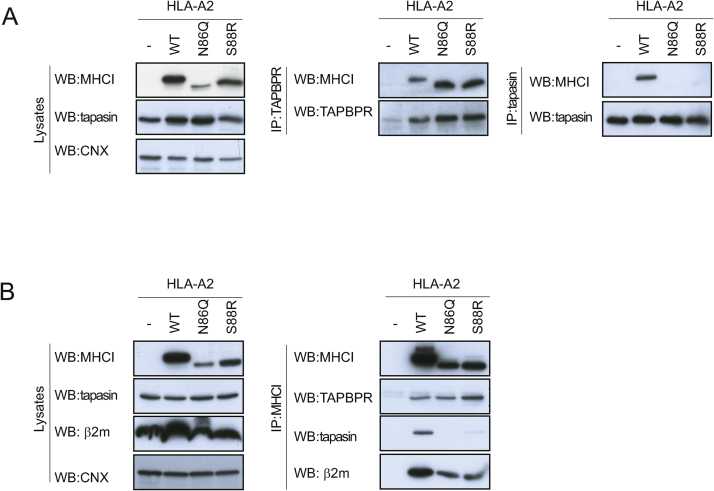


**Figure:**
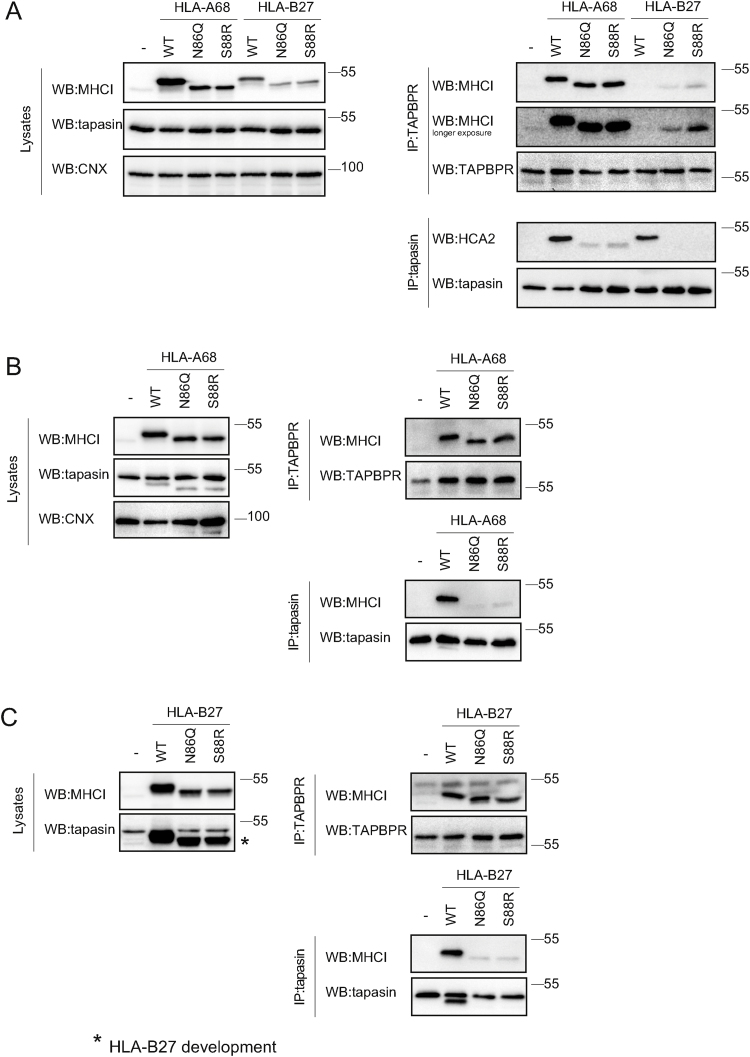


## References

[bib0005] Barbosa J.A., Santos-Aguado J., Mentzer S.J., Strominger J.L., Burakoff S.J., Biro P.A. (1987). Site-directed mutagenesis of class I HLA genes. Role of glycosylation in surface expression and functional recognition. J Exp. Med..

[bib0010] Barnstable C.J., Bodmer W.F., Brown G., Galfre G., Milstein C., Williams A.F., Ziegler A. (1978). Production of monoclonal antibodies to group-a erythrocytes, hla and other human cell-surface antigens - New tools for genetic-analysis. Cell.

[bib0015] Boyle L.H., Gillingham A.K., Munro S., Trowsdale J. (2006). Selective export of HLA-F by its cytoplasmic tail. J. Immunol..

[bib0020] Boyle L.H., Hermann C., Boname J.M., Porter K.M., Patel P.A., Burr M.L., Duncan L.M., Harbour M.E., Rhodes D.A., Skjodt K., Lehner P.J., Trowsdale J. (2013). Tapasin-related protein TAPBPR is an additional component of the MHC class I presentation pathway. Proc. Natl. Acad. Sci. U. S. A..

[bib0025] Braud V.M., Allan D.S., Wilson D., McMichael A.J. (1998). TAP- and tapasin-dependent HLA-E surface expression correlates with the binding of an MHC class I leader peptide. Curr. Biol..

[bib0030] Chen M., Bouvier M. (2007). Analysis of interactions in a tapasin/class I complex provides a mechanism for peptide selection. EMBO J..

[bib0035] Del Cid N., Jeffery E., Rizvi S.M., Stamper E., Peters L.R., Brown W.C., Provoda C., Raghavan M. (2010). Modes of calreticulin recruitment to the major histocompatibility complex class I assembly pathway. J. Biol. Chem..

[bib0040] Dick T.P., Bangia N., Peaper D.R., Cresswell P. (2002). Disulfide bond isomerization and the assembly of MHC class I-peptide complexes. Immunity.

[bib0045] Greenwood R., Shimizu Y., Sekhon G.S., DeMars R. (1994). Novel allele-specific, post-translational reduction in HLA class I surface expression in a mutant human B cell line. J. Immunol..

[bib0050] Harris M.R., Lybarger L., Myers N.B., Hilbert C., Solheim J.C., Hansen T.H., Yu Y.Y. (2001). Interactions of HLA-B27 with the peptide loading complex as revealed by heavy chain mutations. Int. Immunol..

[bib0055] Hermann C., Strittmatter L.M., Deane J.E., Boyle L.H. (2013). The binding of TAPBPR and tapasin to MHC class I is mutually exclusive. J. Immunol..

[bib0060] Hermann C., van Hateren A., Trautwein N., Neerincx A., Duriez P.J., Stevanovic S., Trowsdale J., Deane J.E., Elliott T., Boyle L.H. (2015). TAPBPR alters MHC class I peptide presentation by functioning as a peptide exchange catalyst. Elife.

[bib0065] Howarth M., Williams A., Tolstrup A.B., Elliott T. (2004). Tapasin enhances MHC class I peptide presentation according to peptide half-life. Proc. Natl. Acad. Sci. U. S. A..

[bib0070] Li S., Sjogren H.O., Hellman U., Pettersson R.F., Wang P. (1997). Cloning and functional characterization of a subunit of the transporter associated with antigen processing. Proc. Natl. Acad. Sci. U. S. A..

[bib0075] Lutz P.M., Cresswell P. (1987). An epitope common to HLA class I and class II antigens, Ig light chains, and beta 2-microglobulin. Immunogenetics.

[bib0080] Martayan A., Sibilio L., Setini A., Lo Monaco E., Tremante E., Fruci D., Colonna M., Giacomini P. (2008). N-linked glycosylation selectively regulates the generic folding of HLA-Cw1. J Biol Chem..

[bib0085] Morozov G.I., Zhao H., Mage M.G., Boyd L.F., Jiang J., Dolan M.A., Venna R., Norcross M.A., McMurtrey C.P., Hildebrand W., Schuck P., Natarajan K., Margulies D.H. (2016). Interaction of TAPBPR, a tapasin homolog, with MHC-I molecules promotes peptide editing. Proc. Natl. Acad. Sci. U. S. A..

[bib0090] Neerincx A., Boyle L.H. (2017). Properties of the tapasin homologue TAPBPR. Curr. Opin. Immunol..

[bib0095] Neerincx A., Hermann C., Antrobus R., van Hateren A., Cao H., Trautwein N., Stevanovic S., Elliott T., Deane J.E., Boyle L.H. (2017). TAPBPR bridges UDP-glucose:glycoprotein glucosyltransferase 1 onto MHC class I to provide quality control in the antigen presentation pathway. Elife.

[bib0100] Ortmann B., Copeman J., Lehner P.J., Sadasivan B., Herberg J.A., Grandea A.G., Riddell S.R., Tampe R., Spies T., Trowsdale J., Cresswell P. (1997). A critical role for tapasin in the assembly and function of multimeric MHC class I-TAP complexes. Science.

[bib0105] Parham P., Brodsky F.M. (1981). Partial purification and some properties of BB7.2. A cytotoxic monoclonal antibody with specificity for HLA-A2 and a variant of HLA-A28. Hum. Immunol..

[bib0110] Parham P., Alpert B.N., Orr H.T., Strominger J.L. (1977). Carbohydrate moiety of HLA antigens. Antigenic properties and amino acid sequences around the site of glycosylation. J. Biol. Chem..

[bib0115] Peaper D.R., Wearsch P.A., Cresswell P. (2005). Tapasin and ERp57 form a stable disulfide-linked dimer within the MHC class I peptide-loading complex. EMBO J..

[bib0120] Perosa F., Luccarelli G., Prete M., Favoino E., Ferrone S., Dammacco F. (2003). Beta 2-microglobulin-free HLA class I heavy chain epitope mimicry by monoclonal antibody HC-10-specific peptide. J. Immunol..

[bib0125] Radcliffe C.M., Diedrich G., Harvey D.J., Dwek R.A., Cresswell P., Rudd P.M. (2002). Identification of specific glycoforms of major histocompatibility complex class I heavy chains suggests that class I peptide loading is an adaptation of the quality control pathway involving calreticulin and ERp57. J. Biol. Chem..

[bib0130] Rizvi S.M., Del Cid N., Lybarger L., Raghavan M. (2011). Distinct functions for the glycans of tapasin and heavy chains in the assembly of MHC class I molecules. J. Immunol..

[bib0135] Sadasivan B., Lehner P.J., Ortmann B., Spies T., Cresswell P. (1996). Roles for calreticulin and a novel glycoprotein, tapasin, in the interaction of MHC class I molecules with TAP. Immunity.

[bib0140] Santos-Aguado J., Biro P.A., Fuhrmann U., Strominger J.L., Barbosa J.A. (1987). Amino acid sequences in the alpha 1 domain and not glycosylation are important in HLA-A2/beta 2-microglobulin association and cell surface expression. Mol. Cell. Biol..

[bib0145] Sernee M.F., Ploegh H.L., Schust D.J. (1998). Why certain antibodies cross-react with HLA-a and HLA-G: epitope mapping of two common MHC class I reagents. Mol. Immunol..

[bib0150] Shalem O., Sanjana N.E., Hartenian E., Shi X., Scott D.A., Mikkelson T., Heckl D., Ebert B.L., Root D.E., Doench J.G., Zhang F. (2014). Genome-scale CRISPR-Cas9 knockout screening in human cells. Science.

[bib0155] Stam N.J., Spits H., Ploegh H.L. (1986). Monoclonal-antibodies raised against denatured hla-B locus heavy-chains permit biochemical-characterization of certain hla-C locus products. J. Immunol..

[bib0160] Stam N.J., Vroom T.M., Peters P.J., Pastoors E.B., Ploegh H.L. (1990). HLA-a- and HLA-B-specific monoclonal antibodies reactive with free heavy chains in western blots, in formalin-fixed, paraffin-embedded tissue sections and in cryo-immuno-electron microscopy. Int. Immunol..

[bib0165] Tannous A., Pisoni G.B., Hebert D.N., Molinari M. (2015). N-linked sugar-regulated protein folding and quality control in the ER. Semin. Cell. Dev. Biol..

[bib0170] Teng M.S., Stephens R., Du Pasquier L., Freeman T., Lindquist J.A., Trowsdale J. (2002). A human TAPBP (TAPASIN)-related gene. TAPBP-R. Eur. J. Immunol..

[bib0175] Tiwari R.K., Kusari J., Sen G.C. (1987). Functional equivalents of interferon-mediated signals needed for induction of an mRNA can be generated by double-stranded RNA and growth factors. EMBO J..

[bib0180] Wearsch P.A., Cresswell P. (2007). Selective loading of high-affinity peptides onto major histocompatibility complex class I molecules by the tapasin-ERp57 heterodimer. Nat. Immunol..

[bib0185] Wearsch P.A., Cresswell P. (2008). The quality control of MHC class I peptide loading. Curr. Opin. Cell. Biol.

[bib0190] Wearsch P.A., Peaper D.R., Cresswell P. (2011). Essential glycan-dependent interactions optimize MHC class I peptide loading. Proc. Natl. Acad. Sci. U. S. A..

[bib0195] Williams A.P., Peh C.A., Purcell A.W., McCluskey J., Elliott T. (2002). Optimization of the MHC class I peptide cargo is dependent on tapasin. Immunity.

[bib0200] Yang S.Y., Morishima Y., Collins N.H., Alton T., Pollack M.S., Yunis E.J., Dupont B. (1984). Comparison of one-dimensional IEF patterns for serologically detectable HLA-a and B allotypes. Immunogenetics.

[bib0205] Zhang Q., Tector M., Salter R.D. (1995). Calnexin recognizes carbohydrate and protein determinants of class I major histocompatibility complex molecules. J. Biol. Chem..

[bib0210] Zhang W., Wearsch P.A., Zhu Y., Leonhardt R.M., Cresswell P. (2011). A role for UDP-glucose glycoprotein glucosyltransferase in expression and quality control of MHC class I molecules. Proc. Natl. Acad. Sci. U. S. A..

